# Voltammetric approach for pharmaceutical samples analysis; simultaneous quantitative determination of resorcinol and hydroquinone

**DOI:** 10.1186/s13065-022-00905-y

**Published:** 2022-12-12

**Authors:** Ebrahim Nabatian, Mahdi Mousavi, Mostafa Pournamdari, Mehdi Yoosefian, Saeid Ahmadzadeh

**Affiliations:** 1 Student Research Committee, Kerman University of Medical Sciences, Kerman, Iran; 2 Department of Chemistry, Faculty of Sciences, Shahid Bahonar University of Kerman, Kerman, Iran; 3 Department of Medicinal Chemistry, Faculty of Pharmacy, Kerman University of Medical Sciences, Kerman, Iran; 4 Department of Chemistry, Faculty of Chemistry and Chemical Engineering, Graduate University of Advanced Technology, Kerman, Iran; 5 Pharmaceutics Research Center, Institute of Neuropharmacology, Kerman University of Medical Sciences, Kerman, Iran; 6 Pharmaceutical Sciences and Cosmetic Products Research Center, Kerman University of Medical Sciences, Kerman, Iran

**Keywords:** Resorcinol, Hydroquinone, Voltammetric analysis, Pharmaceutical samples, Modified carbon paste electrode

## Abstract

A simple and precise analytical approach developed for single and simultaneous determination of resorcinol (RC) and hydroquinone (HQ) in pharmaceutical samples using carbon paste electrode (CPE) modified with 1-Ethyl-3-methylimidazolium tetrafluoroborate as ionic liquid and ZnFe_2_O_4_ nanoparticle. A significant enhancement in the peak current and sensitivity of the proposed sensor observed by using modifiers in the composition of working electrode compared to bare CPE which is in accordance with the results obtained from electrochemical impedance spectroscopy investigations. Electrochemical investigations revealed a well-defined irreversible oxidation peak for RC over a wide concentration range from 3.0 µM to 500 µM in 0.1 M phosphate buffer solution (pH 6.0) with the linear regression equations of I_p_ (µA) = 0.0276 *C*_*RC*_ (µM) + 0.5508 (R^2^ = 0.997). The limit of detection and quantification for RC analysis were found to be 1.46 µM and 4.88 µM, respectively. However, the obtained SW voltammograms for simultaneous determination of RC and HQ exhibited a desirable peak separation of about 360 mV potential difference and a satisfactory linear response over the range of 50–700 µM and 5-350 µM with the favorable correlation coefficient of 0.991 and 0.995, respectively. The diffusion coefficient (D) of RC and the electron transfer coefficient (α) at the surface of ZnFe_2_O_4_/NPs/IL/CPE estimated to be 2.83 × 10^− 4^ cm s^− 1^ and 0.76. The proposed sensor as a promising and low-cost method successfully applied for determination of RC in commercial pharmaceutical formulations such as the resorcinol cream of 2% O/W emulsion available on the market with the recovery of 98.47 ± 0.04.

## Introduction

Dihydroxybenzenes as the important phenolic compounds with high toxicity and low degradability suspected of being carcinogens extremely released into the environmental mediums since they used as the chemical intermediate for the synthesis of the variety of pharmaceuticals and other organic compounds such as dyes, photography chemicals, plastics, flavoring agents, antioxidant, rubber, and pesticides. Therefore, they listed as priority pollutants by environmental organizations such as US-EPA and EU [1, 2].

Resorcinol (RC, 1,3-dihydroxybenzene) and hydroquinone (HQ, 1,4-dihydroxybenzene) as two pharmaceutical products extensively used for the treatment of skin diseases. RC commonly applied for acne medication and the treatment of chronic skin diseases such as psoriasis and hidradenitis suppurativa [3, 4]. However, due to its toxic effect at the higher doses, RC disrupts the function of the nervous system which causes acute respiratory problems as well as the endocrine system such as thyroid gland function. On the other hand, HQ as a skin whitening product inhibits the enzymatic pathway of tyrosinase for producing pigment melanin from dopamine [5, 6]. Due to the extraordinary toxicity of HQ at high concentration, it causes nausea, edema of internal organs, headache, dizziness, and even kidney damage in humans [3, 7].

In order to discriminate the two mentioned dihydroxybenzene isomers RC and HQ with similar properties and structure, numerous analytical procedures employed including chromatography [8], fluorescence [9], spectrophotometry [10], fluorometry [3], chemiluminescence [4], and electrochemical methods [3, 6, 7] .

Most of the mentioned instrumental methods are time-consuming, costly; require complicated sample preparation and expert operator which is not suitable for routine analysis. In contrast, the electrochemical techniques received extraordinary attention due to their low cost, rapid response, easy operation, low detection limit, and relatively short analysis time [11, 12]. Recently a few modified electrochemical sensors developed for the simultaneous determination of RC and HQ in biological and pharmaceutical samples [3–7]. However, they suffered from the narrow dynamic concentration range and an undesirable lower detection limit.

Among the modified electrodes, carbon paste electrodes (CPEs) received extraordinary attention due to the advantages of easy preparation and renewability, generous surface chemistry, stable response, wide potential window and low ohmic resistance. In addition to all the benefits mentioned, the use of modifiers that effectively accelerate and facilitates the electron transport between the analyte and the electrode has made the modified carbon paste electrodes a suitable candidate for simultaneous measurement of the analytes by reducing the overpotential required for the electrode reactions [13, 14].

To improve the electrochemical conductivities of bare CPE, room temperature ionic liquid and synthesized nanoparticles namely 1-ethyl-3-methylimidazolium tetrafluoroborate and ZnFe_2_O_4_ used as the modifiers to form a stable carbon paste composite in the current work, respectively. The unique physicochemical characteristics of the mention materials resulted in a better electrochemical response of the modified CPE particularly for the quantitative determination of trace analytes [15, 16]. Ionic liquids (ILs) with remarkable chemical and thermal stability, acceptable electrochemical windows and desirable ionic conductivity properties received considerable attention in modifying the CPEs. ILs provide benefits such as improving the electron transfer rate, sensitivity, and conductivity of the modified CPEs compared to bare CPEs [17]. On the other hand, to provide a larger active surface area with desired catalytic activity for facilitating the electron transport between the analyte and modified CPEs surface, metal nanoparticles extensively applied in the fabrication of electrochemical sensors [18, 19]. Moreover, metal nanoparticles as an efficient catalyst enhanced the electrochemical reactions of electrochemical sensors and biosensors [8, 20–22].

Therefore, herein great attempts have been done to develop a highly selective and sensitive sensor for quantitative determination of trace amount of RC in commercial pharmaceutical formulations available on the market using the proposed modified CPE. To the best of our knowledge, for the first time a square wave voltammetric method developed for simultaneous determination of RC and HQ in the current work. The proposed sensor as a promising and low-cost method successfully applied for determination of RC in commercial pharmaceutical formulations such as the resorcinol cream of 2% O/W emulsion available on the market.

## Experimental

### Chemicals and reagent

Analytical grade resorcinol (RC), iron (III) chloride hexahydrate (FeCl_3_·6H_2_O), zinc (II) chloride (ZnCl_2_), sodium hydroxide (NaOH), sodium bicarbonate (NaHCO_3_), calcium sulfate (CaSO_4_), magnesium nitrate hexahydrate (Mg(NO_3_)_2_·6H_2_O), potassium carbonate (K_2_CO_3_), 1-ethyl-3-methylimidazolium tetrafluoroborate (IL), graphite fine powder extra pure, and extra pure paraffin obtained from Sigma-Aldrich. Glucose, ascorbic acid, phenylalanine, methionine, alanine, valine, isoleucine, urea, and thiourea obtained from Merck. Phosphate buffer solutions (PBS) with the desired pH values prepared using 0.1 M H_3_PO_4_ and 0.1 M NaOH solutions.

### Instruments

The applied electrochemical compartment consisted of a conventional three-electrode system including ZnFe_2_O_4_/NPs/IL/CPE, platinum wire, and Ag/AgCl (3 M KCl) as working, counter, and the reference electrode, respectively. Electrochemical investigations carried out by Autolab PGSTAT204-Metrohm potentiostat/galvanostat programmed and controlled by NOVA 1.11 software and equipped with FRA module for electrochemical impedance spectroscopy studies.

All experiments carried out at room temperature. The pH adjustment performed by a Metrohm pH meter model 827 pH lab (Metrohm AG, Switzerland). To evaluate the morphological aspects of the synthesized ZnFe_2_O_4_ nanoparticle, field emission scanning electron microscopy (FE-SEM) X-Ray Diffraction (XRD), and UV-Vis spectroscopy analysis carried out using TESCAN MIRA3 XMU FE-SEM and Panalytical X’Pert Pro MPD X-Ray Diffraction System, and Optizen 3220 UV spectrophotometer, respectively,

### Nanoparticle synthesis procedure

The aqueous solutions of 0.4 M iron chloride FeCl_3_·6H_2_O, 0.2 M zinc chloride ZnCl_2_ prepared in distilled water. The volume of 25 mL of each solution mixed to each other in Erlenmeyer flask under stirrer 300 rpm condition. Afterward, 25 mL of 3.0 M NaOH solution as the precipitating agent added dropwise to the above solution using burette under same stirrer condition where the gradual formation of precipitate observed. The obtained colloidal solution which synthesized by chemical coprecipitation method, filtered and the pH of the precipitate adjusted at 7.0 by washing with distilled water. Moreover, to improve the nucleation and growth of nanoparticles in the proposed solution, microwave heating and reflux process applied in the current work as follow. 25 mL of aqueous solution containing the collected precipitate placed in the microwave for 30 min at 600 watts. The pH of the solution adjusted at 7.0 by adding required amount of 0.5 M NaOH solution. The obtained precipitate dried at room temperature overnight. Subsequently, the reflux process carried out for the obtained precipitate in the presence of H_2_O:EtOH (1:2) binary solvent for 45 min. finally, the achieved precipitate filtered and its pH adjusted at 7.0 using distilled water and dried at room temperature for 24 h.

### Electrode modification procedure

To prepare the ZnFe_2_O_4_/NPs/IL/CPE, an optimized proportion of 0.1 g synthesized ZnFe_2_O_4_ nanoparticles and 0.9 g graphite powder mixed with abrasion in a mortar. To ensure the uniformity of the resulting mixture, ethyl ether as a highly volatile and ineffective solvent added to the mixture. The mixing process continued until the solvent evaporated completely. Then, an optimized proportion of 0.2 g 1-Ethyl-3-methylimidazolium tetrafluoroborate as ionic liquid and 0.8 g paraffin was added to the mixture dropwise, and after each drop, the mixture mixed with mortar to obtain a uniform paste. An appropriate portion of the prepared paste injected into a glass tube and connected to the electrochemical workstation by a copper wire. To achieve a perfectly flat and uniform surface of the working electrode, the paste pushed by wire and the end of the glass tube polished on a glossy sheet of paper.

## Results and discussion

### Characterization of the synthesized ZnFe_2_O_4_ nanoparticle

To evaluate the successful synthesis of ZnFe_2_O_4_ nanoparticle, FE-SEM, XRD and UV-Vis techniques employed.

**Fig. 1:**
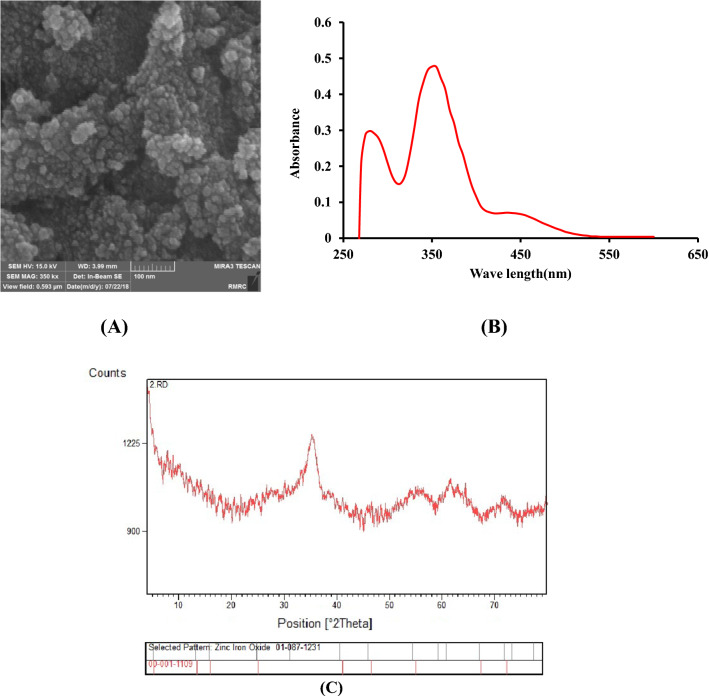
**A** FE-SEM image of ZnFe_2_O_4_ nanoparticles. **B** UV-Vis absorption spectra of ZnFe_2_O_4_ nanoparticles. **C** Representative XRD pattern of ZnFe_2_O_4_ nanoparticles

Field emission scanning electron microscopy (FE-SEM) with high-resolution operating at 15 KeV accelerating voltage applied to investigate the surface details and morphology of the synthesized ZnFe_2_O_4_ nanoparticle. As demonstrated in Fig. 1A, a three-dimensional nanostructure with a high surface area obtained. According to the obtained micrograph, the ZnFe_2_O_4_ nanoparticles exhibit a typical homogeneous morphology with a spherical structure which aggregate to some extent. It concluded that the enhancement in peak current of modified carbon paste electrode attributed to the increase in the active surface area of the working electrode due to the usage of the ZnFe_2_O_4_ nanoparticle.

The XRD analysis performed from 2.0° (2θ) to 80.0° (2θ) and the diffraction data analyzed using PDF2 database. As seen from the XRD patterns of ZnFe_2_O_4_ nanoparticle presented in Fig. 1C, the diffraction peaks at 2θ of 30.06°, 35.45°, 43.03°, 53.54°,57.16°,62.72°,and 73.99° with the calculated d-spacings of 0.297 nm, 0.253 nm, 0.210 nm, 0.171 nm, 0.161 nm, 0.148 nm, and 0.128 nm can be assigned to (220), (311), (400), (422), (511), (440), and (533) reflection planes of the regular spinel cubic structure of ZnFe_2_O_4_ with the space group of Fd3m (JCPDS No. 77–0011), respectively. To calculate the size of the synthesized nanoparticle, the Scherrer equation employed. The average size of 15 nm obtained for ZnFe_2_O_4_ nanoparticle using the peak corresponding to (311) reflection plane.

On the other hand, UV-Vis spectroscopy applied to evaluate the particle size of the synthesized nanoparticle. The absorbance recorded at the wavelength from 250 to 600 nm with 5 nm step size. As seen from the obtained absorption spectra in Fig. 1B, the maximum absorption peak achieved at 350 nm. The average particle size of the synthesized ZnFe_2_O_4_ nanoparticle calculated using the equation expressed as follow [23]:

1 $$Particle\;size\; (nm)={ \left[\left\{\frac{-0.2963+(-40.1970+ \frac{13,620}{{\lambda }_{p}}}{-7.34+ \frac{2418.6}{{\lambda }_{p}}}\right\}\right]}^{2}$$

The calculated particle size was found to be 13.5 nm which is in excellent accordance with the obtained size from FE-SEM and XRD analysis.

### Electrochemical behavior of RC in different pH

**Fig. 2:**
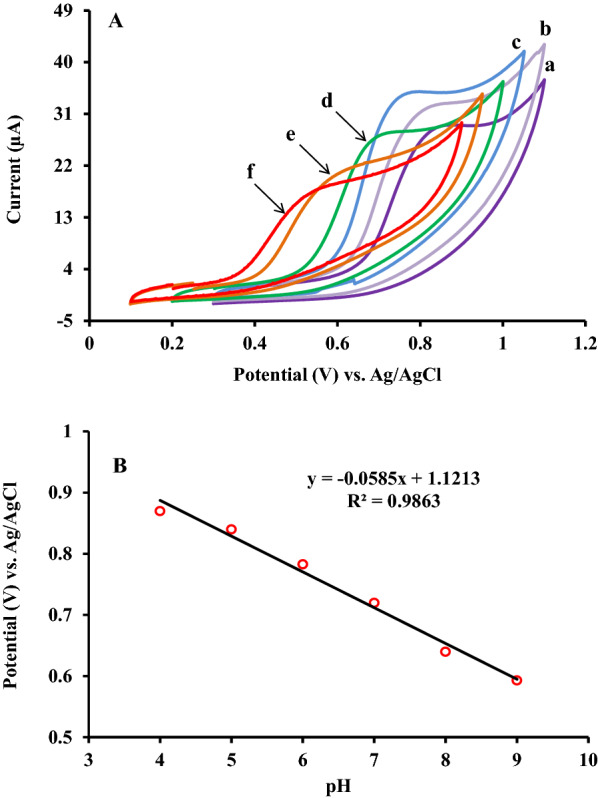
**A** Cyclic voltammetric curves of 500 µM RC on the surface of ZnFe_2_O_4_/NPs/IL/CPE at different pH values of phosphate buffer solution (PBS): 4(a), 5(b), 6(c), 7(d), 8(e), and 9(f). **B** Peak potential dependence on solution pH for RC oxidation on the surface of ZnFe_2_O_4_/NPs/IL/CPE

According to the Nernst equation, the pH of the electrolyte solution and the existence of proton play an important role in the intensity of the oxidation process of electro-active species. Therefore, the effect of solution pH on the electrochemical oxidation of RC investigated. The pH of the electrolyte solution was changed from 4 to 9 using 0.1 M PBS and the oxidation peaks of RC (500 µM) recorded applying ZnFe_2_O_4_/NPs/IL/CPE. The obtained results revealed that the potential of the oxidation peak shifted to less positive values by increasing the solution pH which indicated that the proton involved in the electrocatalytic oxidation of RC (see Fig. 2A). As it is obvious, the value of anodic peak current enhanced by increasing the solution pH from 4 to 6, however, for further increase in solution pH from 6 to 9, a decrease in the value of anodic peak current observed [24]. Accordingly, the optimum solution pH of 6 with the maximum amount of the oxidation current was selected as the ideal buffer solution and applied throughout the current work.

**Scheme 1:**
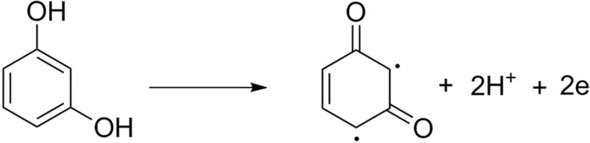
Mechanism of RC electro-oxidation on the surface of ZnFe_2_O_4_/NPs/IL/CPE

By plotting the peak potential (*E*_*p.a.*_ in V) versus the solution pH a straight line with the linear regression equation of E_p_ (V) = − 0.0585 pH + 1.1213 (R^2^ = 0.9863) obtained (see Fig. 2B). By comparing the obtained slope of 0.0585 with the Nernstian slope of 0.0591* m*/*n*, where m and n denote the number of protons participated and electron transferred through the electrochemical reaction, it can be concluded that the number of protons and electrons that involved in the oxidation process of RC are equal. In accordance with the evidence presented above, the Scheme 1 could be suggested as the oxidation mechanism of RC (Scheme 1).

### Improvement of modified CPE electrochemical performance

**Fig. 3:**
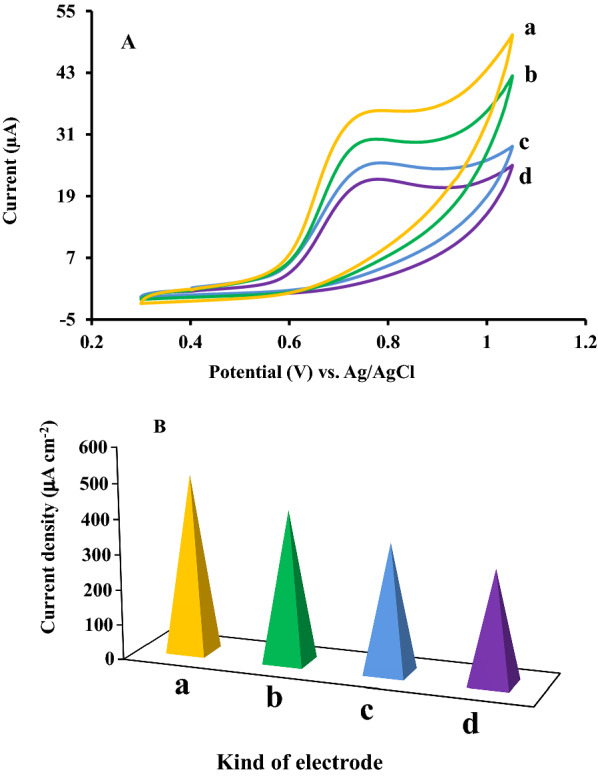
**A** Cyclic voltammetric curves of 500 µM RC in PBS (0.1 M) pH 6 on the surface of (a) ZnFe_2_O_4_/NPs/IL/CPE, (b) IL/CPE, (c) ZnFe_2_O_4_ /NPs /CPE and (d) CPE at scan rate of 100 mV. s^− 1^. **B** The current densities derived from cyclic voltammetric curves at the same electrodes

To investigate the electrocatalytic effect of modifications process on the characteristics performance of the applied carbon paste electrode in the current work, the proposed bare electrode modified over several steps [25]. All investigations conducted with the optimum value of pH solution 6 in 0.1 M PBS at the scan rate of 100 mV s^− 1^ in the presence of 500 µM RC using cyclic voltammetry technique. The RC oxidation peak potential was found to be around 780 mV with the peak current of 22.3 µA by applying the bare CPE. However, optimizing the catalytic ability of CPE ingredients by adding a fraction of nanoparticles and ionic liquid instead of graphite powder and paraffin, respectively, revealed a substantial increase on the surface conductivity of the applied CPE which resulted in enhancement of oxidation current along with shifting the oxidation potential to a more negative value. As seen from Fig. 3A, the overvoltage of RC oxidation process decreased on the surface of CPE, IL/CPE, ZnFe_2_O_4_/NPs/CPE, and ZnFe_2_O_4_/NPs/IL/CPE (curves a–d, respectively). As a result, the recorded RC oxidation peak on the surface of ZnFe_2_O_4_/NPs/CPE exhibited significant oxidation current of 35.5 µA around 765 mV.

Furthermore, the surface current density of the mentioned electrodes calculated from the obtained related oxidation peak current and demonstrated in Fig. 3B. The obtained results revealed that the applied modifiers resulted in enhancement of active surface area of proposed electrodes which is in accordance with the conducted electrochemical impedance spectroscopy investigations [26].

### Electrochemical impedance characterization

Electrochemical impedance spectroscopy (EIS) as a dominant diagnostic tool applied for characterization of the interface structure of electrolyte solution/electrode and the electrode surface nature. Herein, EIS as an experimental technique used for describing the observed changes in characteristic performance of carbon paste electrode throughout its modification process using ZnFe_2_O_4_/NPs and 1-Ethyl-3-methylimidazolium tetrafluoroborate as the binder.

**Fig. 4:**
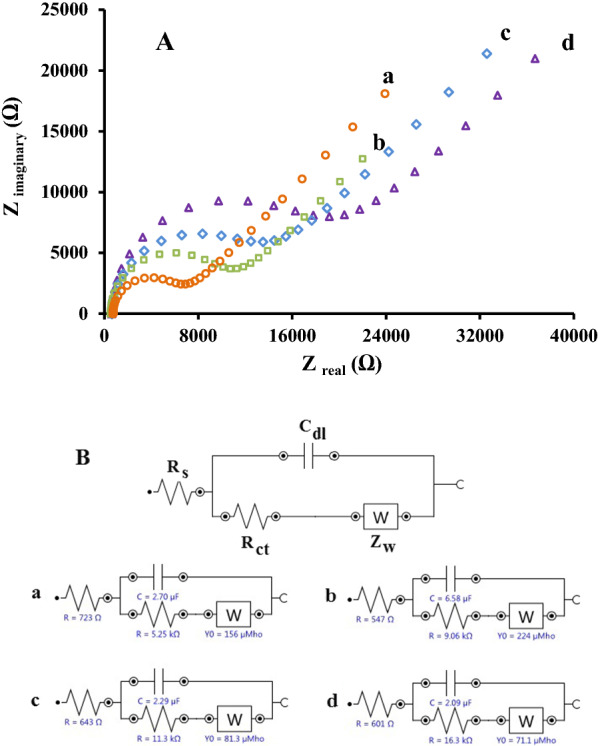
**A** Nyquist diagrams of (a) ZnFe_2_O_4_/NPs/IL/CPE, (b) IL/CPE, (c) ZnFe_2_O_4_ /NPs /CPE and (d) CPE. Conditions: 500 µM RC in PBS (0.1 M) pH 6, over the frequency range of 0.1 to 100,000 Hz. **B** Corresponding equivalent circuits

The EIS investigations conducted over the frequency range of 10^− 2^ to 10^5^ Hz. As seen from Fig. 4A, the obtained Nyquist plots for CPE, ZnFe_2_O_4_/NPs/CPE, IL/CPE and ZnFe_2_O_4_/NPs/IL/CPE revealed that by modifying the CPE, the diameter of the semicircle portion at higher frequency which corresponds to the charge transfer limited process decreased and indicated that the electron transfer resistance on the surface of the proposed electrodes gradually diminished and accordingly, the highest charge transfer rate observed at the surface of ZnFe_2_O_4_/NPs/CPE.

The linear part of the Nyquist plot at lower frequency represent the limited diffusion process. The values of the charge transfer resistance for CPE, ZnFe_2_O_4_/NPs/CPE, IL/CPE and ZnFe_2_O_4_/NPs/IL/CPE found to be 16.30 kΩ, 11.30 kΩ, 9.06 kΩ, and 5.25 kΩ, respectively.

The equivalent circuit of Fig. 4B obtained by modeling the impedance data of Nyquist plots in the term of an electrical circuit. The proposed equivalent circuit constituted of R_s_ denotes the electrolyte solution resistance in series with the parallel circuit of Z_f_ and C_dl_ denote the Faradaic impedance and the double layer capacitance, respectively. Z_f_ composed of two parameters including R_ct_ and Z_w_ denote the charge transfer resistance and the Warburg impedance, respectively.

### Characterization of scan rate effect

**Fig. 5:**
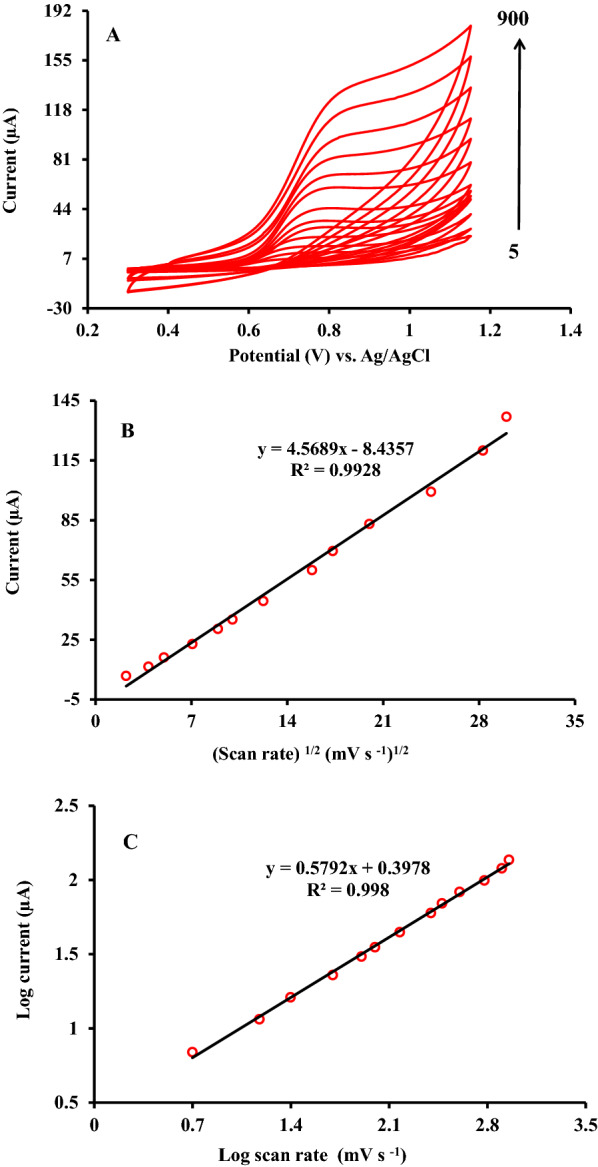
**A** Cyclic voltammetric curves of the ZnFe_2_O_4_/NPs/IL/CPE at different potential scan rates of 5, 15, 25, 50, 80, 100, 150, 250, 300, 400, 600, 800 and 900 mV s^− 1^ in PBS (0.1 M) pH 6 containing 500 µM RC. **B** Peak current dependence on the square root of scan rate for RC oxidation on the surface of ZnFe_2_O_4_/NPs/IL/CPE. **C** Relationship between the logarithm of peak potential and logarithm of the potential scan rate

To investigate the nature of the RC oxidation and its kinetic parameters at ZnFe_2_O_4_/NPs/IL/CPE, the relationship between the potential scan rate and peak current over the range of 5-900 mV/s studied in the presence of 500 µM of RC, using cyclic voltammetry. As seen from the voltammograms in Fig. 5A increasing the potential scan rate resulted in a gradual increase in the oxidation peak current. However, the potential of the oxidation peak shift towards more positive values which indicated that the electro-oxidation of the RC was irreversible.

As seen from Fig. 5B, by investigating the relationship between the anodic peak current intensity (I_p_) vs. potential scan rate (ν) and the square root of potential scan rate (ν^1/2^), it was found that a satisfactory linear relationship between I_p_ and ν^1/2^ with a correlation coefficient of 0.993 observed which confirmed that the electrode process of RC oxidation controlled by diffusion mechanism at ZnFe_2_O_4_/NPs/IL/CPE [27]. The obtained correlation equation expressed as below:

2 $${{\text{I}}_{\text{p}}}\left( {\mu {\text{A}}} \right) = {4.5689}{{\text{n}}^{{1}/{2}}}({{\text{mV}}^{{1}/{2}}}{{\text{s}}^{ - {1}/{2}}}) - {8.4357}\left({\text{R}^{2}} = {0.9928} \right)$$

Alternatively, by plotting the Log I_p_ versus Log ν, the electrode process regarding mass transport mechanism can be specified. It is known that the slope values around 0.5 denote the redox process controlled under the diffusion step. However, the slope values around 1.0 indicated that the redox processes ruled by adsorption. The obtained result from Fig. 5C was in accordance with the recommended mechanism in the previous section.

**Fig. 6:**
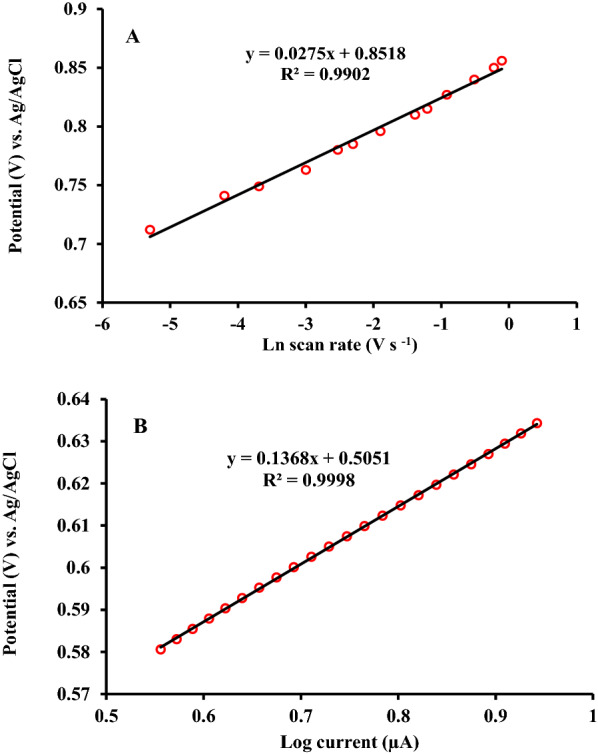
**A** Nicholson and Shain’s plot of oxidation peak potential vs. the Naperian logarithm of different potential scan rates of 5, 15, 25, 50, 80, 100, 150, 250, 300, 400, 600, 800 and 900 mV s^− 1^ in PBS (0.1 M) pH 6 containing 500 µM RC. **B** Tafel’s plot of oxidation peak potential vs. the logarithm of the peak current for the electro-oxidation of 500 µM RC on the surface of ZnFe_2_O_4_/NPs/IL/CPE at the scan rate of 25 mV s^− 1^ in PBS (0.1 M) pH 6 containing 500 µM RC.

To determine the electron transfer coefficient (α) of the irreversible oxidation process of RC, the relationship between the oxidation peak potential (E, V) and the Naperian logarithm of the potential scan rate (Ln ν, V s^− 1^) investigated. The obtained plot in Fig. 6A, revealed an adequate linear relationship with the regression equation expressed as follow:

3 $${{\text{E}}_{\text{p}}}\left( {\text{V}} \right) = {0.0275}\,{\text{Lnn}}\left( {{{\text{Vs}}^{-1}}} \right) +{0.8518} \left( {{\text{R}}^{2}} = {0.9902} \right)$$

According to the equation proposed by Nicholson and Shain correspond to the graph of E (V) vs. Ln ν (V.s^− 1^) expressed as follow, the value of electron transfer coefficient (α) of 0.766 calculated from the slope of the obtained plot which is m/2, where m is equal to RT/[(1–α)n_α_F] .

4 $${{\text{E}}_{\text{p}\text{a}}=\text{E}}^{0}+\text{m}[0.78+\text{ln}\left({\text{D}}^{\frac{1}{2}}{{\text{k}}_{\text{s}}}^{-1}\right)-0.5\text{ln}\text{m}]+\left(\frac{\text{m}}{2}\right)\text{l}\text{n}({\upnu })$$

where E_p.a._, E^0^, ν, and k_s_ denote the oxidation peak potential, formal potential, potential scan rate, and the electron transfer rate constant, respectively. Assuming the number of electrons involved through the electro-oxidation process (n) is equal to 2. Furthermore, R, T, and F are equal to 8.314 J mol^− 1^ K^− 1^, 298 K, and 96,485 C mol^− 1^, respectively.

Additionally, via the data derived from the raising part of the RC oxidation curve (current vs. potential), the Tafel plot was developed [28]. As seen from Fig. 6B, a linear relationship between peak potential (*E*_*p.a.*_) and the logarithm of the peak current (*Log I)* with the satisfactory correlation coefficient of 0.999 observed. The respective equation expressed as below:

5 $${{\text{E}}_{\text{p}}}\left( {\text{V}} \right) ={0.136}{\text{Log I}}\left({\mu {\text{A}}} \right) + {0.5051}\left({{{\text{R}}^{2}} ={0.9998}} \right)$$

Herein, alternatively, the value of electron transfer coefficient (α) can be calculated from the slope of the Tafel plot which is equal to 2.303RT/[(1–α)n_α_F]. The electron transfer coefficient value was found to be 0.783 which is in accordance with the obtained value for (*α)* from to the graph of E (V) vs. Ln ν (V s^− 1^).

### Chronoamperometric investigation

**Fig. 7:**
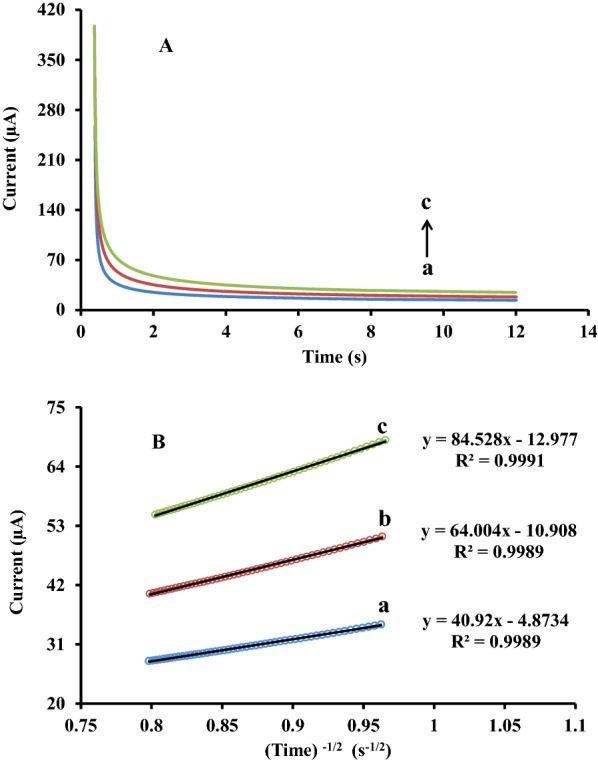
**A** Exponential single potential-step chronoamperometic curves of 300 (a), 500 (b) and 700 (c) µM RC in PBS (0.1 M) pH 6. **B** Cottrell’s plot of oxidation peak current vs. the minus square roots of time for 300 (a), 500 (b) and 700 (c) µM RC in PBS (0.1 M) pH 6

Chronoamperometry technique employed to assess the diffusion coefficient (D) of RC at the surface of ZnFe_2_O_4_/NPs/IL/CPE. The working electrode potential set at 1000 mV vs. the reference electrode. As seen in Fig. 7A, chronoamperograms recorded for three concentrations of 300, 500, and 700 µM resorcinol in 0.1 M phosphate buffer solution (pH 6.0).

Through the data derived from the mass transport limited part of the chronoamperogram curves, by plotting the peak current (I_p_) versus the minus square roots of time (t^− 1/2^) the Cottrell plots obtained. As demonstrated in Fig. 7B, oxidation currents has a linear relation with t^− 1/2^ at all three mention concentrations which confirmed that the mass transport mechanism at the surface of working electrode controlled under the diffusion step from the bulk solution toward the ZnFe_2_O_4_/NPs/IL/CPE surface [29]. The average value of the diffusion coefficient was found to be 2.83 × 10^− 4^ cm^2^s^− 1^ by substituting the slopes of Cottrell plots and other parameters in the Cottrell equation.

### Analytical performance characterization

The characteristics performance of the fabricated ZnFe_2_O_4_/NPs/IL/CPE investigated with regard to several operating parameters including linearity of the proposed sensor response over the wide concentration range of RC, limit of detection (LOD) and quantification (LOQ), repeatability and reproducibility, lifetime and the effect of interferences presence.

**Fig. 8:**
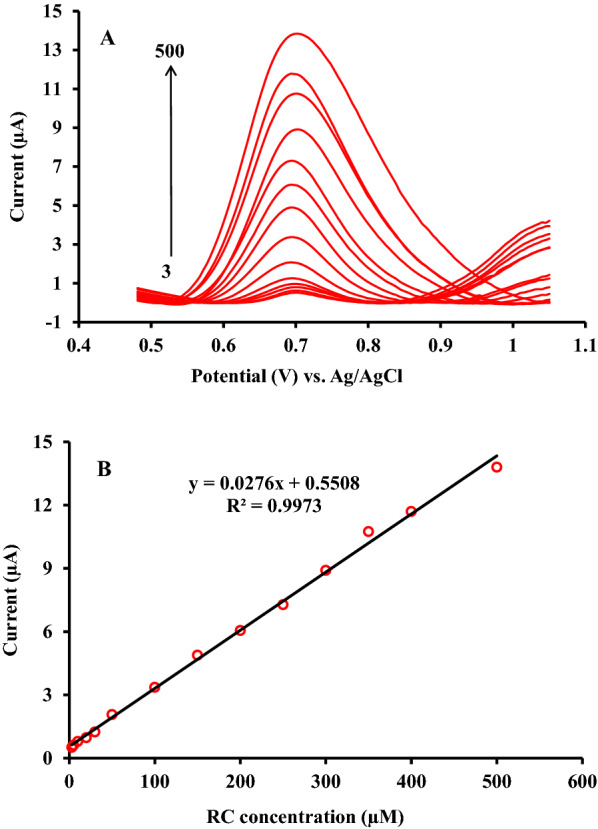
**A** Square wave voltammetric curves for successive additions of RC into PBS (0.1 M) pH 6 including RC concentrations of 3, 5, 10, 20, 30, 50, 100, 150, 200, 250, 300, 350, 400, and 500 µM on the surface of ZnFe_2_O_4_/NPs/IL/CPE. **B** Typical calibration curve corresponding to RC additions up to 500 µM

The square wave voltammetry (SWV) with lower background current compare to cyclic voltammetry adopted for the determination of RC over a wide concentration range in 0.1 M phosphate buffer solution (pH 6.0). As seen from Fig. 8, a linear relationship between oxidation peaks current (*I*_*p*_) and the concentration of RC over the range from 3.0 µM to 500 µM with the satisfactory correlation coefficient of 0.997 observed.

The observed deviation from the linear response at higher concentration probably attributed to the diffusion of RC or accumulation of the undesired oxidation products on the surface of the proposed electrode. The respective linear regression equations expressed as below:

I_p_ (µA) = 0.0276 *C*_*RC*_ (µM) + 0.5508 (R^2^ = 0.9973) (6).

The value of the limit of detection (LOD) for the proposed electrode calculated according to the definition of 3S_b_/m, where S_b_ as the standard deviation of peak current derived from 10 measurements of the blank solution (S_b_=1.348 × 10^− 8^) and m is the slope of the linear calibration plot (m = 0.0276). The lower detection limit of the proposed sensor was found to be 1.46 µM RC. Furthermore, the value of the limit of quantification (LOQ) according to the definition of 10S_b_/m was found to be 4.88 µM RC by SWV employing ZnFe_2_O_4_/NPs/IL/CPE.

To evaluate the accuracy and precision of the fabricated ZnFe2O4/NPs/IL/CPE, the repeatability and reproducibility of the proposed electrode assessed. The repeatability investigated with five successive scans in one day and five scans during five days using the same electrode. The examined solutions contain 5.0 µM RC. The obtained results revealed satisfactory repeatability with the relative standard deviation of ± 1.33 and ± 2.70, respectively. On the other hand, for reproducibility studies, five different electrodes were used only once each and the obtained results indicated good reproducibility of the proposed sensor with the relative standard deviation of ± 3.41.

Moreover, to evaluate the stability of the response, ZnFe_2_O_4_/NPs/IL/CPE immersed in an aqueous solution and applied for the quantitative determination of RC known concentrations in various samples. Assessment of the obtained results indicated that the electrode revealed stable response within 180 min and afterward the background current increased. The observed behavior is possibly related to the leakage of 1-butyl-3-methylimidazolium tetrafluoroborate from the modified carbon paste which increased the roughness of the fabricated electrode. The obtained results showed that ZnFe_2_O_4_/NPs/IL/CPE has acceptable repeatability and reproducibility with a satisfactory stability.

### Resorcinol and hydroquinone simultaneous electrochemical determination

**Fig. 9:**
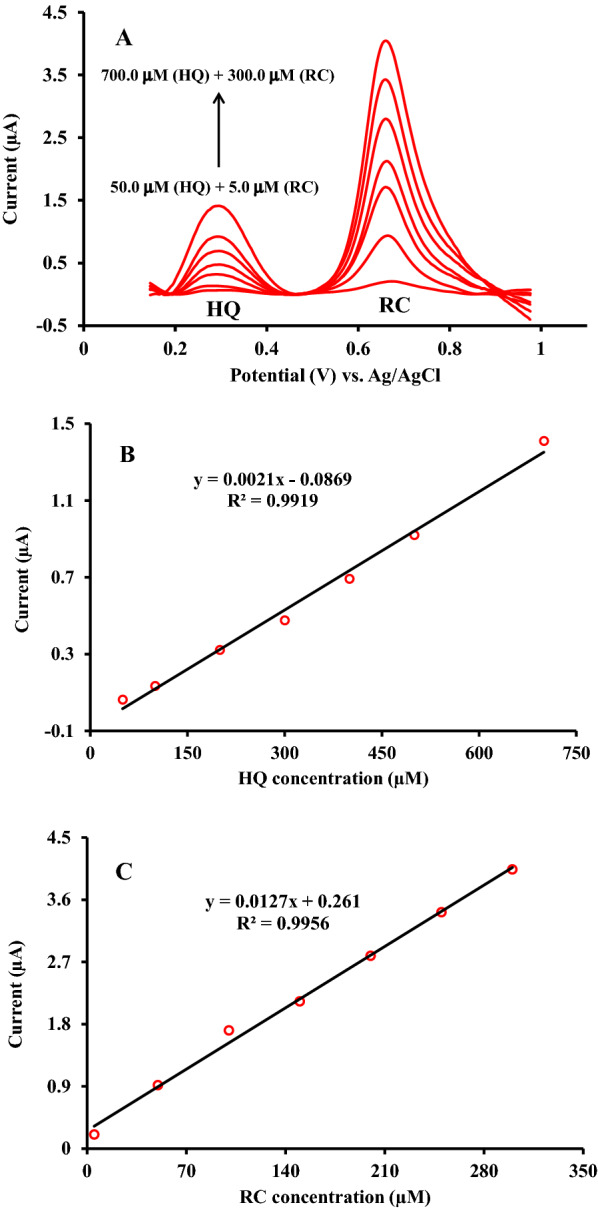
**A** Square wave voltammetric curves for simultaneous additions of HQ and RC into PBS (0.1 M) pH 6; from inner to outer including HQ and RC concentrations of 50.0 + 5.0, 100.0 + 50.0, 200.0 + 100.0, 300.0 + 150.0, 400.0 + 200.0, 500.0 + 250.0, and 700.0 + 300.0 µM, respectively. **B** and **C** Typical calibration curves corresponding to HQ and RC additions up to 700 and 300 µM, respectively

In order to provide a voltammetric approach for simultaneous determination of RC and HQ in pharmaceutical products, SWV technique with high sensitivity and capability of oxidation peaks separation employed in the current work. The SWVs plot recorded by the simultaneous change of RC and HQ concentrations over a wide range in 0.1 M phosphate buffer solution (pH 6.0). As seen in Fig. 9, two separate and highly intense oxidation peaks at 290 mV and 650 mV related to HQ and RC achieved. A satisfactory linear relationship between oxidation peak current (*I*_*p*_) and the concentration of HQ and RC over the range of 50–700 µM and 5-350 µM with the favorable correlation coefficient of 0.991 and 0.995, respectively, observed.

It is apparent that at the surface of ZnFe_2_O_4_/NPs/IL/CPE a desirable peak separation with the potential difference of about 360 mV (vs. Ag/AgCl reference electrode) for HQ and RC obtained. The obtained results revealed that the proposed electrode could be applied for the concurrent determination of RC and HQ in the presence of each other without significant deviation in the electrochemical response.

### Determination of RC in the presence of coexisting interfering species

Further studies carried out to evaluate the ability of the proposed sensor for discriminating between the desired target of RC and the potential interfering species which present in real samples such as pharmaceutical formulations and biological fluids. Herein, the effect of interfering species including various kind of electrolytes, amino acids, and sugar on the characteristic performance of ZnFe_2_O_4_/NPs/IL/CPE investigated in the presence of 50 µM RC under the optimized experimental condition (see Table 1). It is noteworthy to mention that the tolerance limit of the proposed electrode defined as the maximum amount of interfering compounds which resulted in a peak current deviation more than 5.0% for determination of RC compared to square wave voltammograms of RC solution alone.

**Table 1:** The effect of some coexisting substances on the determination of 50 µM resorcinol (n = 3)

Species	Tolerance limits (W/W: n-fold excess weight of interference vs. resorcinol)
HCO_3_^−^, Ca^2+^, Mg^2+^, Na^+^, K^+^, SO_4_^2−^, CO_3_^2−^, NO_3_^−^	500
Glucose	500
Ascorbic acid	200
Phenylalanine, methionine, alanine, valine, isoleucine	700
Urea, thiourea	400

The obtained results revealed that the oxidation peak current of RC deviated from the tolerance limit in the presence of more than 500-fold excess of common electrolytes including Ca^2+^, Mg^2+^, Na^+^, K^+^, SO_4_^2−^, CO_3_^2−^, NO_3_^−^, and HCO_3_^−^. However, the presence of glucose and ascorbic acid in the 500-fold and 200-fold excess concentration had no interference with RC detection, respectively. On the other hand, the oxidation peak current of RC evaluated in the presence of 700-fold excess of common amino-acids including phenylalanine, methionine, alanine, valine, and isoleucine which used in the biosynthesis of proteins. It found that the mention concentration did not show interference in the quantitative determination of RC.

Lastly, the presence of some substances which need to excrete from the body such as urea evaluated on the characteristic performance of the proposed electrode. The results showed that the ZnFe_2_O_4_/NPs/IL/CPE response deviated from the tolerance limit in the presence of more than 400-fold excess of urea and thiourea.

### Analysis of the pharmaceutical sample

In order to evaluate the performance of the fabricated ZnFe_2_O_4_/NPs/IL/CPE for precise determination of RC in the real samples, the resorcinol cream of 2% O/W emulsion available on the market studied. To prepare the real sample of resorcinol cream for analysis, firstly, RC which is a weakly acidic compound must be extracted from the cream by the following method.

1 g of the cream carefully weighed and completely dissolved in 9 mL of distilled water. To extract the RC from the obtained milky color diluted base, 2 g of NaCl salt added for salting out process. Subsequently, the pH of the obtained emulsion adjusted around 12.5 by adding NaOH 0.1 M, which is 3 units higher than the pK_a_ for RC. Accordingly, the RC compound converted into the ionized and hydrophilic form which could easily extract from the lipophilic components of the obtained emulsion and transferred into the aqueous phase. The obtained mixture centrifuged for 20 min at 4000 rpm. 100 µL of the supernatant phase transferred into the electrochemical cell and diluted up to 10 mL by the phosphate buffer solution (0.1 M, pH 6.0). As seen from Table 2, the commercial resorcinol cream contains % 1.9 (w/w) resorcinol in the O/W emulsion.

**Table 2:** Determination of RC in resorcinol cream of 2% O/W emulsion available on the market by the proposed sensor

Resorcinol cream of 2% O/W emulsion	Obtained oxidation peak current*(µA)	Measured concentration (mg)	Recovery	% W/W Resorcinol
1 g	1.81	19.12	98.47 ± 0.04	% 1.9

### Analytical performance comparison of the proposed sensor with previous works

The analytical performance of ZnFe_2_O_4_/NPs/IL/CPE for simultaneous determination of RC and HQ compared to the other reported sensors. As seen from Table 3, the lower detection limit of the proposed electrode was better than some reported graphene-based cases [3, 4]. However, the carbon paste electrode in the current work is much cheaper than the mention electrodes. On the other hand, the present sensor revealed a wider dynamic linear range compared to most of the summarized reported sensors [3, 5, 6]. It can be concluded that the present electrode is either comparable or superior compared to the other reported sensors for simultaneous determination RC and HQ.

**Table 3:** Comparison of the analytical performance of the proposed sensor for the simultaneous determination of RC and HQ with other electrochemical sensors found in the literature

Electrode (specificity)	Technique (specificity)	pH	Linearity and Range (µM)		LOD (µM)		Refs.
			HQ	RC	HQ	RC	
MWCNT–SH@Au–GR/GCE ^a^	DPV	7	54.5–1250.5	43.5–778.5	4.17	7.80	[3]
P-rGO ^b^	DPV	7	5–90	5–90	0.08	2.62	[30]
Nafion/MWCNTs/CDs/MWCNTs ^c^	DPV	7	1–200	1–400	0.07	0.15	[7]
MEA-MWCNTs ^d^	Amperometry	5.40	1–100	6–100	0.3	0.6	[3]
PANI/MnO_2_ ME ^e^	DPV	7	0.2–100	0.2–100	0.13	0.09	[5]
Graphene doped CILE ^f^	DPV	5	10–400	1–170	1.8	0.4	[6]
ZnFe_2_O_4_/NPs/IL/CPE	SWV	6	50–700	3.0-500	23.5	1.46	Current work

^a^Gold nanoparticle–graphene nanohybrid bridged 3-amino-5-mercapto-1,2,4-triazole-functionalized multiwall carbon nanotubes

^b^ Porous reduced graphene oxide

^c^ Nafion/multi-walled carbon nanotubes/carbon dots/multi-walled carbon nanotubes

^d^ multielectrode array modified with multiwall carbon nanotubes

^e^ Polyaniline (PANI) nanofibers / MnO_2_ modified electrode

^f^ Graphene Doped Carbon Ionic Liquid Electrode

## Conclusion

The excellent electro-catalytic performance of RC and HQ at the surface of ZnFe_2_O_4_/NPs/IL/CPE which was not susceptible to common interferences provided a new promising approach for simultaneous determination of trace amount of RC and HQ in pharmaceutical samples using square wave voltammetry technique. The SW voltammograms revealed two well-defined separated oxidation peaks with a desirable peak separation and a satisfactory linear response over a wide concentration range of RC and HQ. The developed modified carbon paste electrode showed a considerable improvement in the kinetics of the electron transfer with an excellent reproducible analytical performance which indicated that the proposed sensor could be applied successfully for routine analysis.

## Data Availability

Adequate and clear descriptions of the applied materials and tools are provided in the materials and method section of manuscript. In addition, the obtained data is clearly justified by mentioning the figures and tables in the manuscript.
